# Cross-Cultural Comparison of the Espresso Protocol Repeatability

**DOI:** 10.3390/foods14040593

**Published:** 2025-02-11

**Authors:** Jisoo Choi, Jeehyun Lee, Edgar Chambers

**Affiliations:** 1 Department of Food Science and Nutrition, College of Human Ecology, Pusan National University, Busan 46241, Republic of Korea; qqeyfe@gmail.com; 2 Center for Sensory Analysis and Consumer Behavior, Kansas State University, Manhattan, KS 66502, USA; eciv@ksu.edu

**Keywords:** coffee, expert, sensory characteristic, check-all-that-apply, CLUSCATA, homogeneity, cross-culture

## Abstract

The Espresso Protocol (TEP) was used to assess the quality of coffee beans through espresso extraction incorporating a sensory approach. TEP includes overall quality evaluation and attribute evaluation using check-all-that-apply (CATA). This study aims to evaluate the repeatability of TEP when used by experts and to compare cross-cultural assessments to determine its applicability across different countries with diverse coffee cultures. Experts with over three years of experience in the coffee industry from five countries—France (n = 7), India (n = 12), Italy (n = 7), the Republic of Korea (n = 10), and the USA (n = 10)—participated in our study. The experiment was conducted in three replications using eight different single-origin coffee samples over two or three consecutive days. Cluster analysis using CATA data was performed to verify the repeatability of individual participants in the characterization of espresso samples, revealing that most participants were repeatable in their three-time evaluations. Moreover, a significant homogeneity index demonstrated a high degree of similarity in the sensory characteristics used by experts from each country, although cultural differences were observed in the terminology used to describe coffee. In conclusion, the repeatability of individual experts and the reliability of TEP were successfully demonstrated. However, some differences in sensory evaluations were noted across cultures; these were likely influenced by differences in the use of terminology, which emphasizes the need for training in the coffee lexicon.

## 1. Introduction

One of the most widely consumed beverage is coffee, with a record consumption of 176.6 million bags of coffee in 2021/22 [1]. There is also a growing interest in flavor components that have an important role in marketing the product [2]. Brewing and extraction methods vary across geographic, cultural, and social contexts, as well as individual preferences. Furthermore, coffee is a complex beverage that contains over 1000 compounds contributing to its distinctive flavor and aroma [3]. Espresso is a brewed beverage produced by extracting ground coffee using high-pressure hot water. It is commonly used as the base for various coffee-based beverages [4,5,6]. The quality of espresso can be influenced by several factors, including the coffee variety, blending formulations, grind size, water temperature, and the inherent quality of the coffee itself [7,8].

To evaluate coffee quality in the coffee industry, the cupping method was usually used to measure the sensory properties of coffee. However, some authors have shown that cupping scores vary among cuppers and that there is no correlation between the cupping scores of importers and exporters [2]. The Specialty Coffee Association of America (SCAA) introduced a protocol in 1984 to define the sensory quality of coffee. This protocol is detailed and adaptable to standardization, meeting the needs of producers [2,8]. However, recent concerns have highlighted several limitations of the cupping method. While cupping is widely recognized as a reliable method, it is not suitable for all situations [9]. The cupping committee of SCAA (2005) stated that the purpose of sensory evaluation is (1) to identify actual sensory differences between samples, (2) to describe the taste of the samples, and (3) to determine product preference. In the past, the value of coffee was often determined by the preferences of the cupper through cupping [10]. Since 2020, the SCA has recognized the need to evolve cupping protocols. While recent protocols emphasize quality scoring, they fall short in capturing descriptive information. With the increasing importance of descriptive evaluation, the SCA proposed a new cupping format to collect such information, which was previously lacking. This includes a descriptive evaluation for objective flavor assessment and an emotional evaluation based on overall quality impressions [11]. As a result, the SCA introduced the Coffee Value Assessment (CVA) as the new cupping standard in 2024 [12].

Similarly, in a prior study on TEP [13], ‘The Espresso Protocol’ (TEP) was used to evaluate espresso, including sensory methods such as check-all-that-apply, intensity rating, and just-about-right scale. The early versions of TEP provided a basic framework for assessing espresso, but they had limitations in fully describing the complexity of sensory characteristics.

Furthermore, cultural differences may influence the evaluation results in this study. Kim (2015) reviewed various cross-cultural studies, noting that differences observed in consumer preferences or perceived characteristics are primarily attributable to cultural factors rather than inherent individual differences [14]. As such, cultural differences between countries participating in this study could affect the results. Participants may be reluctant to use negative descriptors and/or extreme values and may have difficulties with translation. In addition, it is essential to recognize and account for cultural customs, manners, religious beliefs, knowledge, and attitudes toward products [15]. To date, most coffee studies have merely compared differences based on elements such as roasting level [4,16], storage [17,18], brewing methods [2,5,19], serving temperatures [20,21], and geographic origin [22,23] of brewed coffee. Furthermore, there have only been a few cross-cultural studies on coffee, where consumers’ coffee purchase and consumption behavior were compared between Chinese and Swedish university students [24], and TEP usage was compared between the United States or Canada (US/CA) and Australia or New Zealand (AU/NZ) [13].

The objectives of our study were (1) to determine the repeatability of the modified TEP, and (2) to identify cross-cultural differences between five countries (France, India, Italy, the Republic of Korea, and the USA) in evaluating espresso using TEP.

## 2. Materials and Methods

### 2.1. Samples

Eight coffee bean types sold wholesale in the U.S. were selected based on prior knowledge, origin, the quality of coffee beans, and the price range of these coffee samples (Table 1). One sample, called Pre-Packaged in our study, is a product that has already been roasted at the medium level and sold, while the other seven samples—Colombia, Ethiopia, Brazil, Honduras, Costa Rica, Guatemala, and Brazil Cerrado—were green beans from each country of origin. A total of eight coffee bean varieties were shipped with the alphabetical labels A through G, each containing 3 KG (6.6 lb); the Pre-Packaged was a bag of 1.5 KG (3.3 lb) of roasted coffee labeled H.

#### 2.1.1. Sample Preparation

In each country, a professional roaster and a barista were recruited for sample preparation. Additionally, experts participating in espresso evaluation were prevented from sample preparation because sample information may cause bias.

The first step of sample preparation was the roasting of the seven green bean samples. As the quantity of coffee beans to be roasted is small (3 KG), the roaster had to use a small batch or large sample roaster (e.g. Probatino, Bellwether Series 2, HB600 or 2 KG, etc.). The roaster was free to determine the appropriate roasting level for each sample considering the country-of-origin information. Although not all countries used the same roast level, most were roasted at a light-medium or medium level. The professional roaster provided details about roasting profiles used in coffee beans, such as roast time, roast level, peak temperature, and other information to describe the roast (Supplementary Table S1). Then, each coffee bean sample was divided into three new bags, one for each replication. The bags used for repacking were provided and labeled with the letters A to H instead of coffee origin, with three-digit random codes that were different for each replication. Approximately 1.1 lb (0.5 KG) of coffee per bag was packed for the three sample bags, with an additional ‘Excess Coffee’ bag for extra coffee in case of problems. Roasting was completed at least 3 days before, but not more than 10 days before the evaluation. The exception to the roasting was India, where roasting was done 20 days before the evaluation due to outsourcing the roasting.

The grinding and brewing were the last step of sample preparation, taken care of by a trained barista. To meet the grind specifications required by TEP, each country had to use a commercial quality grinder, as factors such as grinder type, burr design, and the age and usage (wear) of the burrs all affect the outcome. Also, to meet the brewing standards (Table 2), espresso machines had to be of commercial quality with essential features, including (1) the ability to measure and adjust brewing temperature (setting temperature with a PID, which stands for a Proportional-Integral-Differential controller; a thermostat is optimal), (2) the ability to measure and adjust pump pressure (a rotary pump is optimal), (3) consistent brew temperature throughout the brew cycle, and (4) the use of water that meets the water standards specified in this study (Table 3). The espresso machine had to undergo temperature and pressure checks, and slight adjustments to the grinder settings were required for each sample, prompting the professional barista to repeatedly monitor the coffee preparation process.

In addition, professional baristas were guided to provide samples in accordance with the extraction guidelines of this study. They served each sample promptly for evaluation to maintain the temperature of each serving. For the coffee dose, a range of 15–17 g was selected as the optimal range for the test standard, as it produces a concentrated sensory result suitable for evaluation and meets the preparation standard.

#### 2.1.2. Sample Evaluation Procedure

The eight coffees were evaluated three times each, i.e., three replications. The sample serving orders were balanced, and experts in each country evaluated them in the same order due to constraints in sample preparation for espresso equipment settings. Each sample evaluation took approximately 20 to 30 min to taste and evaluate. Eight to sixteen espresso samples were evaluated in the session, depending on the testing country. France, Italy, and India evaluated the samples over two consecutive days. In France, 16 samples and 8 samples were evaluated in each session, Italy evaluated 12 samples per day; and in India, on the first day, two sessions were held in the afternoon and in the evening, where 8 samples were evaluated in each session. On the next day, one session was held to evaluate the third replication of the 8 samples. Thus, in India, a total of three evaluation sessions were held over two consecutive days. The other countries (the Republic of Korea and the USA) evaluated eight samples per day for three consecutive days. Although these numbers of samples might seem large for typical sensory descriptive studies [25], in Issanchou (2018) [26], it is suggested that evaluating more than 10 samples per session should be avoided, as it may lead to sensory fatigue. Additionally, Kemp (2018) recommended performing 2 to 4 repetitions of the analysis for statistical reliability [27].

For each sample evaluation, one cup of espresso (70–90 mL) was served. Experts evaluated the prepared samples following five steps: (1) On serving the espresso sample, observe the crema and evaluate it; (2) Place a demitasse spoon at the bottom of the cup, stir the espresso back and forth, and then inhale the aroma with the nose over the cup five times and evaluate the aroma; (3) Wait for one minute, then take the first sip to evaluate the flavor and body/mouthfeel; (4) Wait about 30 s, then take a second sip to evaluate the basic taste, continuity, aftertaste, and holistic/hedonic, and then evaluating balance/amplitude and defects; (5) When the espresso has cooled to room temperature, take a third sip, check its stability, and confirm the evaluations. Finally, the questionnaire was completed by providing an open-ended comment for the coffee sample or TEP, evaluating the overall quality score, and checking the willingness to use it as an espresso.

### 2.2. The Espresso Protocol^TM^ (TEP)

TEP is a coffee quality evaluation tool that aims to allow users to quantify and indicate the characteristics and attributes of espresso coffees in an efficient, precise, and repeatable way across panelists and geographies. In our study, TEP is composed of questions in the order of appearance during espresso experiences using multiple check-all-that-apply questions, two 100-point scale questions, several “check-one” questions, two yes/no questions, two expository text questions, and a section for open-ended comments. More details about the questionnaires are shown in Table 4. An online survey tool, Qualtrics^XM^ (Seattle, WA, USA), was used to execute TEP. The evaluation was carried out using personal hand-held mobile devices. Once experts scanned the QR code with the electronic device, they were directed to the corresponding questionnaires. In each replication, experts were given a placemat with the QR code and sample code displayed on it.

To evaluate aroma, flavor, tactile impact, basic tastes, and overall impact, TEP is a questionnaire developed using the World Coffee Research Sensory Lexicon [28]. The initial TEP [13] used the 5-point JAR scale in the protocol, but the questionnaires in this current study were revised by adding more sensory characteristics used in CATA and adding them as drop-down options. The composition of the TEP used in previous studies can be found in Table 1 of Kim (2025) [13]. The same 13 sensory characteristic terms (descriptions) were used in the categories of ‘Aroma’ and ‘Flavor’, and these 13 terms have a more detailed drop-down menu (Table 4). For example, if one selects the description of ‘Fruity’, ‘Nutty’, and ‘Sweet’ in the category of ‘Aroma’, then one will see a screen that selects the detailed description of each of the items. The same logic applies to the ‘Flavor’ category.

### 2.3. Participants

The Espresso Protocol (TEP) participants were composed of a total of 46 experts from France (n = 7), India (n = 12), Italy (n = 7), the Republic of Korea (n = 10), and the USA (n = 10), who currently resided in the designated countries (Table 5). Experts who evaluated coffee using the TEP in this study were recruited as adults over 19 years of age, with at least 3 years of experience in the coffee industry, no caffeine sensitivity, and were not pregnant or lactating.

Before the experiment, the experts who would evaluate the coffee samples were provided with the WCR Sensory Lexicon and the evaluation guidelines, but no separate training sessions were conducted. On the day of the experiment, in order to ensure a smooth online survey process, a detailed explanation of the survey flow and evaluation methods was given to the participants prior to the coffee evaluation, ensuring they had a clear understanding of the procedure. Additionally, the Coffee Taster’s Flavor Wheel (2016) from the Specialty Coffee Association (SCA) and World Coffee Research (WCR) was provided for reference during the evaluation process.

### 2.4. Data Analysis

CLUSCATA was conducted using CATA sensory characterization data to determine whether replications from individual experts were grouped together and whether experts were divided into separate clusters. The homogeneity index of each country was confirmed by the results of CLUSCATA. Cochran’s Q test was performed to verify the frequency of CATA terms and significant differences between eight samples in each evaluation category. Correspondence analysis (CA) was carried out using the same CATA data. The results of CA visually represented the relationship between samples and characteristics, and the similarity of sensory perception was also compared across samples in different countries using RV-coefficients. One-way ANOVA with Tukey post-hoc tests (*p* < 0.05) was conducted to examine the differences in the overall quality of samples evaluated in each country. Two-way ANOVA with Tukey post-hoc tests (*p* < 0.05) was performed to determine which countries and samples were statistically significantly different. CLUSCATA, Cochran’s Q test (performed in CATA data analysis), and RV-coefficients were analyzed using XLSTAT software (version 2024.1.0; Addinsoft Inc., New York, NY, USA). Correspondence analysis, One-way ANOVA, and Two-way ANOVA were analyzed using SAS software (version 9.4; SAS Institution Inc., Cary, NC, USA).

## 3. Results

### 3.1. Repeatability of TEP

CLUSCATA is a method designed to segment subjects such that the homogeneity within a cluster is maximized. It is based on the same principles as CATATIS, which calculates group mean data with down-weighted subjects [29,30]. Building on this, our study sought to verify that the results of TEP were evaluated consistently through CLUSCATA, which we interpret as ensuring reliable evaluation data from experts. The data from each country were the sensory attributes selected in the CATA portion of the study. In XLSTAT, the class number selection was automatically maintained to ensure more accurate cluster analysis, and the integration of classes obtained through the layering algorithm was applied [31]. The homogeneity of the experts in our study, together with the repeatability of TEP, was verified. Based on the data evaluated by the participants, both the homogeneity of individuals and cross-cultural consistency can be confirmed.

#### 3.1.1. Verification of Homogeneity in Expert Evaluation Through CLUSCATA

Table 6 lists the experts grouped together for each of the five clusters. Participant numbers were used as follows: 101–110 for the USA, 201–208 for Italy (excluding 202 due to missing data), 301–310 for the Republic of Korea, 401–412 for India, and 801–807 for France. The four-digit numbers are identifiers used to distinguish the evaluation data, with each number representing one of the three evaluation sessions per participant.

Data from each individual expert’s three replications were grouped into the same cluster. For example, in Cluster A, the first, second, and third replications of expert 302 (1302, 2302, 3302) were grouped in the same cluster, showing consistency for that expert. Overall, five clusters of CATA terms were identified. For Korean experts, two distinct clusters emerged: Clusters A and B. However, the other four countries formed three mixed clusters. This suggests that the Republic of Korea experts, although generally consistent within themselves, use notably distinct sensory terminology compared to those of the other countries.

**Figure 1:**
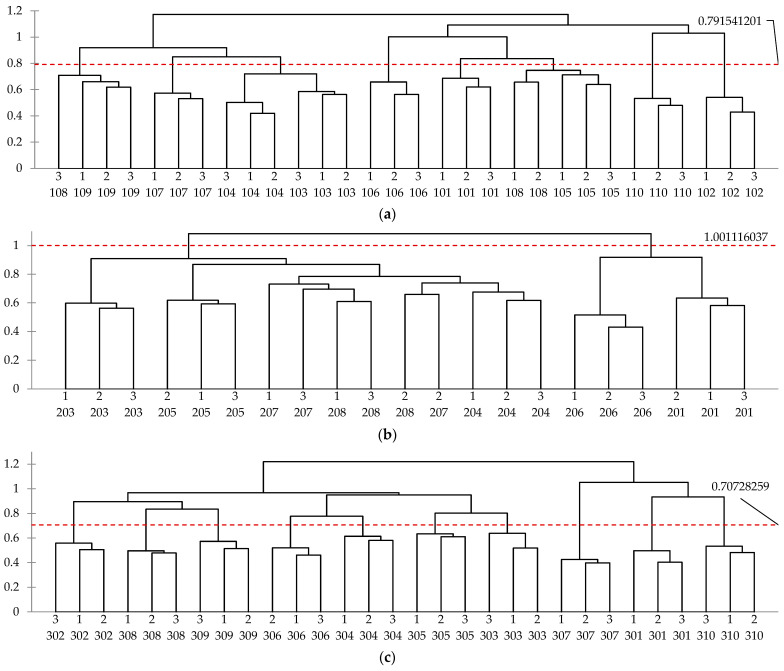

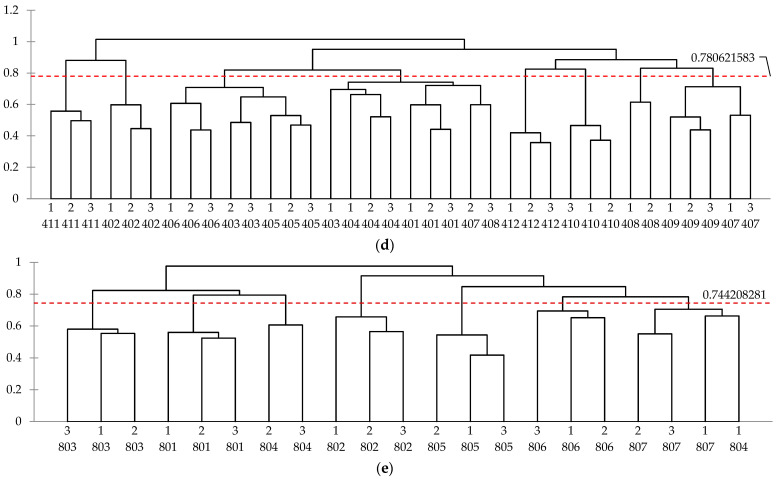
Dendrograms of CLUSCATA results ^1^ for each country based on sensory characteristics. Results of (**a**) USA; (**b**) Italy; (**c**) Republic of Korea; (**d**) India; and (**e**) France. ^1^ The horizontal axis, D (delta), represents the value reflecting the increase in internal heterogeneity when clusters merge.

Figure 1 presents the dendrogram of CLUSCATA results from each country. It was confirmed that the experts’ evaluations were consistent, as the individual data from three replications were generally grouped into individual clusters. In the USA, as shown in Figure 1a, 9 out of 10 experts showed repeatability. In Italy, Figure 1b, 5 out of 7 experts showed repeatability.

In the Republic of Korea, shown in Figure 1c, the homogeneity of individual evaluations was clearly demonstrated, as clusters were formed for each of the 10 participants. Among them, participants 307 and 301 exhibited the highest homogeneity, with scores of 0.7 or higher. In India, as indicated in Figure 1d, 9 out of 12 experts showed repeatability. In France, as shown in Figure 1e, most experts formed a single cluster except for expert 804, leading to 6 out of 7 experts showing repeatability.

In many studies, when CLUSCATA is conducted, very low in similarity and atypical responses are often excluded from the analysis, and on average, approximately one-third of responses are classified as “K + 1 clusters” or “noise clusters” [32]. It has been demonstrated that when atypical respondents were excluded, the homogeneity index of clusters increased [30]. The preceding studies [30] found that the homogeneity index improved to 0.482 and 0.407 as the K + 1 cluster was excluded. However, in this study, there were no data considered atypical respondents; thus, none were excluded as the K + 1 cluster, indicating a good level of homogeneity across all five countries regardless of cross-cultural differences. Although some participants did not show consistent evaluation tendencies across all three evaluations, CLUSCATA analyses showed that most participants evaluated repeatedly.

#### 3.1.2. Comparison of Homogeneity Index by Countries

CLUSCATA calculated the homogeneity index, which results from verifying the similarity of CATA responses among experts. The homogeneity index for each group indicates the similarity in their use of sensory characteristics. Table 7 shows that the global homogeneity index for all evaluation data (n = 138) is 0.364. In particular, the homogeneity index was found to increase as the clusters were further subdivided in each country, suggesting that the terms used within each country were more similar. A recently published chocolate study found homogeneity indices of 0.550 and 0.538 in the results after excluding the K + 1 cluster, with the lowest homogeneity index being 0.402 when evaluated by consumers [30]. In our study, a homogeneity index of 0.5 or higher was observed in four countries, with the exception of Italy (Table 7). In addition, the number of terms provided in TEP was much higher, which can reduce the homogeneity index. In contrast to the findings where the homogeneity index became more pronounced after the removal of atypical respondents [32], our study found that no evaluation data were classified as “K + 1”. This indicates that there were no data negatively impacting the homogeneity index.

Moreover, in Figure 1, the horizontal axis, *D* (delta), represents the value reflecting the increase in internal heterogeneity when clusters merge and should be kept as small as possible [29]. The values on the y-axis represent the distance or difference between clusters as they are merged, facilitating an initial analysis of the clustering level of the data. Specifically, a larger *D* value indicates a greater difference between clusters, while a smaller value suggests higher similarity. This pattern is consistent with the homogeneity index presented in Table 7. The *D* values increase in the following order: Republic of Korea, France, India, USA, and Italy, which corresponds to the same ranking observed in the homogeneity indices.

### 3.2. Cross-Cultural Comparison

#### 3.2.1. Cross-Cultural Comparison of RV Coefficients for Sample Configurations

Table 8 presents the results of comparing sample configurations across five countries based on the CA of sensory characteristics assessed using the CATA method. The closer an RV coefficient is to 1, the more similar the sample and attribute configurations are [33,34,35]. The eight samples were classified using CATA (Check-All-That-Apply) terms that exhibited slight differences by country. The sample configurations in the first and second dimensions of CA for the RV coefficient were higher than 0.7 and significant for five out of ten comparisons. An acceptable degree of similarity was observed in half of the comparisons, despite potential cultural differences (e.g., preference, consumption tendency, type of response, etc.).

There are varying perspectives on the stability of the RV coefficient, and it is important to assess its significance and adjust the threshold according to the required precision [36,37,38]. For instance, an RV value of 0.85 is considered indicative of ‘good test-retest repeatability’ [39], while RV coefficients ranging from 0.65 to 0.71 have been reported as reflecting adequate agreement between consumers and trained panels [40]. Considering these values, five out of ten comparisons in our study were statistically significant, suggesting that the RV coefficients are stable.

**Figure 2:**
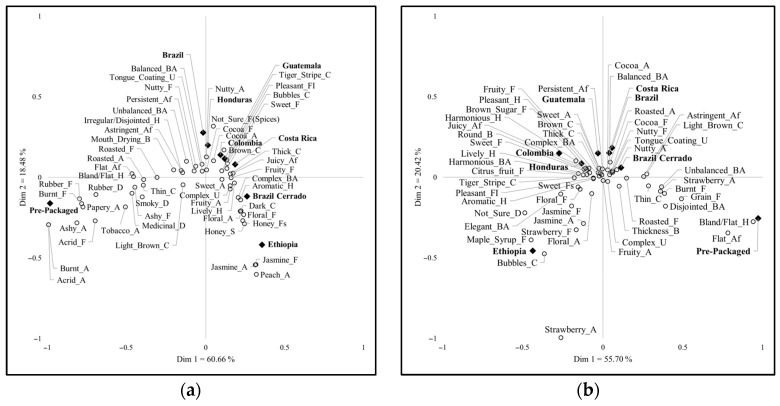

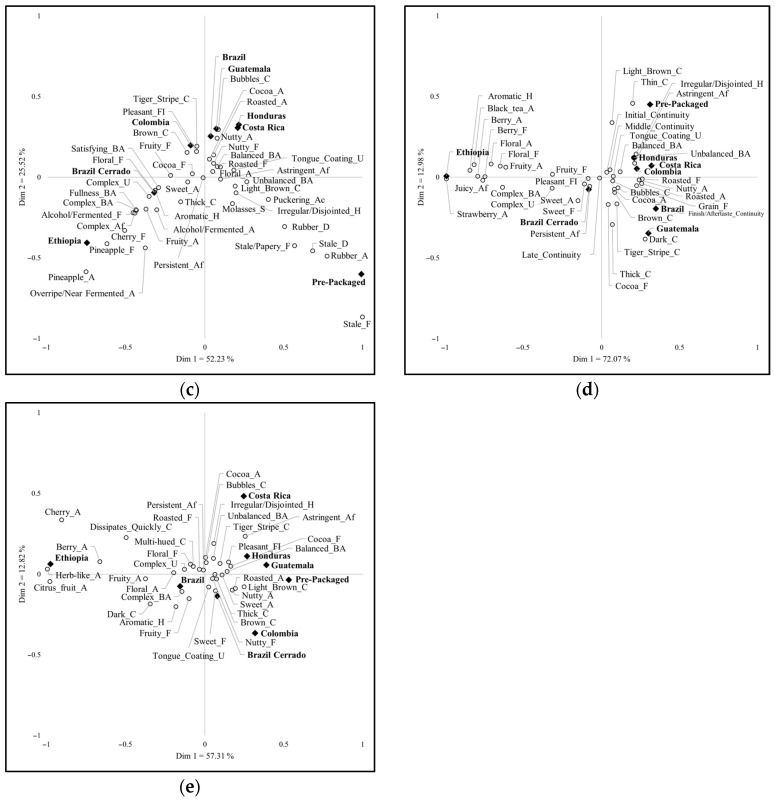
Correspondence analysis ^1^ in each country: (**a**) France; (**b**) India; (**c**) Italy; (**d**) Republic of Korea; (**e**) USA.; ^1^ Empty circles (○) indicate attributes; Filled rhombuses (♦) indicate samples.

Figure 2 presents a visualization of the correspondence analysis (CA) used in the comparison. It must be noted that the proximity of characteristics to each other does not indicate a direct relationship, as this is not represented in the layout [41]. However, the proximity of terms to a sample typically does indicate that some of those terms may be found at higher levels in those closer samples than in other samples. Cochran’s Q test was performed to identify the significant differences between the sample frequencies for each attribute [42], and the results were used to explain CA biplots.

#### 3.2.2. Difference Between Country and Sample on Overall Quality

Table 9 shows the results of a One-way ANOVA on the overall quality score, based on a 0–100 point scale, evaluated in three replicates separately for five countries.

By country, there were significant differences in the samples from all countries except the USA. A common trend emerged across all countries, with Ethiopia being rated as having the highest overall quality and Pre-Packaged coffee as the lowest overall quality. The Pre-Packaged coffee sample had been roasted much earlier before the evaluation, which might have influenced the lower quality score. These common results suggest that the quality evaluation of these two samples was not influenced by cross-cultural factors. However, the rankings of the other six samples varied slightly between countries, which is believed to reflect cultural differences, such as consumer preferences in each country.

**Figure 3:**
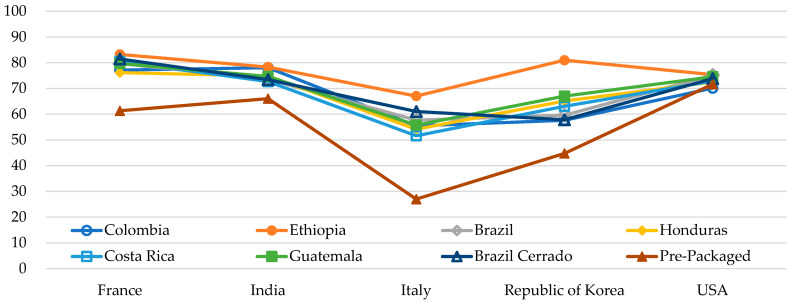
Cross-cultural comparison of significant interaction between eight samples and overall quality scores (*p* < 0.0001).

Figure 3 shows significant interactions of the overall quality score (0–100) between countries and samples (*p* < 0.0001).

The USA did not distinguish between coffee samples, whereas Italy and the Republic of Korea employed a broader range of the scale to differentiate samples based on the overall quality score. At first glance, this data suggests that the experts in most countries were more discriminating among samples than those in the USA. However, because almost all the USA experts were consistent in their evaluations, the data may suggest that the USA group of experts simply found all samples to be of equal quality. Differences in quality evaluation are exemplified by the ‘Brazil Cerrado’ and ‘Brazil’ coffee samples, which differed between countries, likely reflecting what is accepted as good quality coffee in each country.

## 4. Discussion

Analysis of the homogeneity index of CATA data has not been performed frequently so far, and prior research on this topic is limited. Although consumers’ chocolate characterization using CATA research was one of few studies that examined the homogeneity index [30], we report here that experts in our study resulted in higher homogeneity scores without excluding atypical data. In fact, the homogeneity index from a couple of countries was the highest reported thus far.

No atypical data were found in our study, meaning that all expert evaluation data were analytically significant. Although homogeneity among the data was relatively low in Italy, this suggests not only a difference in the use of sensory characteristics but also indicates that these differences may be influenced by the role of the expert in the coffee industry. Based on demographic information from Italy, four out of seven participants were working as roasters. The remaining individuals in Italy included a researcher, a technician, and a staff member. This suggests that it may have been relatively new for them to evaluate coffee using the sensory characteristics or terms that describe coffee. In contrast, nine out of ten Republic of Korea participants took on barista roles, with additional responsibilities in other roles. Only one participant served as a roaster. The Republic of Korea exhibited the highest homogeneity index among the five countries, with the data forming a single cluster, reflecting much higher homogeneity in using terms to describe specific coffee samples. This suggests that homogeneity in using sensory characteristics may be influenced by the participants’ roles.

Although various factors, such as roasting degree, evaluation environment, extraction ratio, extraction yield, and water quality, differed across countries, this present study focused on validating the repeatability of TEP despite these variations. However, there were some differences in the terminology used to describe and assess the samples across countries. Regarding the overall quality score, there was a common trend of evaluation by country. Ethiopia was evaluated as having the highest overall quality coffee, while Pre-Packaged was rated as having the lowest overall quality. These two samples were evaluated similarly across all five countries, suggesting that the quality evaluation for these two samples was not influenced by cross-cultural factors. However, it was not possible to rule out the possibility that the other six samples were evaluated with slight differences in ranking. Additionally, in the open-ended questions asking about the preferred country of origin, Ethiopia was the most frequently mentioned, followed by Kenya, Colombia, India, and Panama (Geisha). Other origins mentioned included various regions and varieties, such as Africa, Bourbon, Brazil, Cidra, Congo, Costa Rica (Villa Sarchi), Ecuador, Honduras, Indonesia, Rwanda, and the USA. Based on these responses, it can be inferred that the overall higher quality score for the Ethiopian coffee, even when tested in a blind study, was likely influenced by the evaluators’ familiarity with and preferences for those sensory characteristics.

In this study, we expanded the terminology for describing sensory characteristics when evaluating coffee using TEP, allowing for a more effective and diverse description of the coffee than in previous TEP studies [13]. This approach enables a more detailed tracking of the characteristics of espresso extraction as required by buyers and producers. Although the roasting degree was not identical across countries, the individual reproducibility and repeatability of TEP were sufficiently demonstrated, meeting the objectives of this study.

The experts were ‘untrained’ in the specific terms used in this study, although they had received training in the overall concepts of the WCR lexicon [28] and the coffee flavor wheel [43]. That makes them more ‘trained’ than consumers, but, like consumers, experts may face challenges in evaluating coffee due to its complex flavor compounds. Several studies have demonstrated that meaningful results can be achieved through targeted training for coffee evaluation [9,44,45,46,47,48]. By developing educational tools tailored to consumers’ level of understanding of the sensory lexicon, the use of TEP can be made more effective, allowing the coffee industry to better align its products with consumers’ perspectives, ultimately enhancing consumers’ espresso experience.

## 5. Limitations

There are several limitations in this study. First, the number of participants varied by country. Although we initially planned to recruit 10 experts from each country, the actual number of participants ranged from 7 to 12, depending on the circumstances in each country. Additionally, differences in terminology understanding and usage across countries and individuals impacted the repeatability and homogeneity of the evaluations. It is believed that training on sensory characteristics, using a standardized framework such as the WCR sensory lexicon, is necessary to improve the homogeneity and clarity of espresso evaluations. Finally, participants were unfamiliar with the protocol and, thus, it would have taken some time for familiarization. Repeated use of TEP in future studies should help increase the homogeneity of the evaluation results.

## 6. Conclusions

In conclusion, our study successfully demonstrated the repeatability of TEP, a protocol for evaluating espresso with experts. Despite variables such as roasting level, extraction recipes, and water quality varying across countries, the homogeneity of sensory characteristics and individual repeatability were high in most countries, yielding meaningful results. Although there were some differences in the terminology used to describe the samples, it could be improved through training on the sensory lexicon. Notably, the Ethiopia and Pre-Packaged samples were not influenced by cross-cultural factors, considering both the overall quality score and CATA characterizations, while differences in terminology and the overall quality score ranking of the other six samples were observed.

In future studies, the effect of coffee lexicon training preceding TEP use should be considered. In addition, determining whether consumers can use TEP would be important because TEP could be utilized as a communication tool between the coffee industry and their direct consumers’ perspectives on quality coffee.

Additionally, by classifying roles within the coffee industry and identifying the specific factors that contribute to differences between these roles, utilizing TEP can generate meaningful data that would also offer valuable information to the coffee industry.

## Figures and Tables

**Table 1:** Coffee bean information.

Label	Origin	Information			
		Region	Farmer	Processing Method	Variety
A	Colombia	Nariño	Aponte Community	Washed	Caturra
B	Ethiopia	Guji and Yirgacheffe	Smallholders	Washed	Heirloom
C	Brazil	Sul di Minas	-	Natural	Cauai
D	Honduras	-	-	-	-
E	Costa Rica	Tarrazu, San Jose	Tarrazu Smallholders	Washed	Caturra, Catuai
F	Guatemala	Huehertenango	Smallholders	Washed	Caturra, Marsellesa, Catuai, and Bourbon
G	Brazil Cerrado	Cerrado Mineiro	Hidetoshi Alberto Otaguri	Extended Fermentation	-
H	Pre-Packaged	Up to nine origins	Varies	Mixed	Arabica

**Table 2:** Brewing standards.

Parameters	
Brewing Pressure	9 Bar
Temperature	92–94 °C (198–201 °F)
Filter Basket Volume	15–17 g ± 1 g) to make two servings (Use the filter basket that matches the size of the dose, either 15 g or 17 g.)
Liquid Weight	70–90 mL, two servings of 35–45 mL each
Extraction Time	25 s ± 3 s
Extraction Ratio	50–55% coffee weight to extraction time

**Table 3:** Water standards ^1^.

Characteristic	Target	Acceptable Range
Odor	Clean, Fresh/Odor Free	Clean, Fresh/Odor Free
Color	Clear Color	Clear Color
Total Chlorine	None	None
TDS	150 mg/L	75–250 mg/L
*Calcium Hardness*	*50–175 ppm CaCO_3_* *(4 grains or 68 mg/L)*	*50–175 ppm CaCO_3_* *(1–5 grains or 17 mg/L–85 mg/L)*
*Total Alkalinity*	*40 ppm*	*At or near 40–70 ppm*
pH	7.0	6.0–8.0
Sodium	10 mg/L	At or near 10 mg/L

^1^ The specifications are based on those set by the SCAA Standards Committee (Water Standards Version:21NOV2009A, by the Specialty Coffee Association of America) for use in brewing specialty coffee with a few modifications for TEP based on industry recommendations (shown in italics).

**Table 4:** The Espresso Protocol^TM^ (TEP) questionnaire composition.

Evaluation Category	Scale	Terms in CATA
		**A. Description of the espresso**
Crema	CATA	None, Hole in crema, Dark, Brown, Light Brown, Off Color, Multi-hued, Tiger Stripe, Persistent, Dissipated Quickly, Splotchy, Bubbles, Thick, Thin
Aroma, Flavor ^1)^	CATA	**Fruity** ^4)^, **Floral** ^4)^, **Nutty** ^4)^, **Cereal** ^4)^, **Cocoa** ^4)^, **Sweet** ^4)^, **Earthy** ^4)^, **Roasted** ^4)^, **Spices** ^4)^, **Vegetative** ^4)^, **Stale/Papery** ^4)^, **Chemical** ^4)^, **Alcohol/Fermented** ^4)^
Fruity ^1)^	CATA	**Citrus fruit** ^4)^, **Lemon** ^4)^, **Grapefruit** ^4)^, **Orange** ^4)^, **Lime** ^4)^, **Berry** ^4)^, **Strawberry** ^4)^, **Raspberry** ^4)^, **Blackberry** ^4)^, **Dried Fruit** ^4)^, **Raisin** ^4)^, **Prune** ^4)^, **Other Fruit** ^4)^, **Apple** ^4)^, **Pear** ^4)^, **Peach** ^4)^, **Grape** ^4)^, **Cherry** ^4)^, **Pomegranate** ^4)^, **Coconut** ^4)^, **Pineapple** ^4)^, Other ^2)^, Not Sure ^3)^
Floral ^1)^	CATA	**Black tea** ^4)^, **Jasmine** ^4)^, **Rose** ^4)^, **Chamomile** ^4)^, Other ^2)^, Not Sure ^3)^
Nutty ^1)^	CATA	**Peanuts** ^4)^, **Almond** ^4)^, **Hazelnut** ^4)^, Other ^2)^, Not Sure ^3)^
Cereal ^1)^	CATA	**Grain** ^4)^, **Malt** ^4)^, Other ^2)^, Not Sure ^3)^
Cocoa ^1)^	CATA	**Cocoa** ^4)^, **Chocolate** ^4)^, **Dark Chocolate** ^4)^, Other ^2)^, Not Sure ^3)^
Sweet ^1)^	CATA	**Sweet** ^4)^, **Molasses** ^4)^, **Maple Syrup** ^4)^, **Brown Sugar** ^4)^, **Caramelized** ^4)^, **Honey** ^4)^, **Vanilla** ^4)^, Other ^2)^, Not Sure ^3)^
Earthy ^1)^	CATA	**Musty/Earthy** ^4)^, **Meaty/Brothy** ^4)^, **Animalic** ^4)^, **Moldy/Damp** ^4)^, **Smoky** ^4)^, **Phenolic** ^4)^, Other ^2)^, Not Sure ^3)^
Roasted ^1)^	CATA	**Acrid** ^4)^, **Brown roast** ^4)^, **Burnt** ^4)^, **Ashy** ^4)^, **Tobacco** ^4)^, **Pipe Tobacco** ^4)^, Other ^2)^, Not Sure ^3)^
Spices ^1)^	CATA	**Brown spice** ^4)^, **Pepper** ^4)^, **Nutmeg** ^4)^, **Clove** ^4)^, **Cinnamon** ^4)^, **Anise** ^4)^, Other ^2)^, Not Sure ^3)^
Vegetative ^1)^	CATA	**Hay-like** ^4)^, **Herb-like** ^4)^, **Beany** ^4)^, **Under-ripe** ^4)^, **Green** ^4)^, **Dark Green** ^4)^, **Peapod** ^4)^, **Olive Oil** ^4)^, **Raw** ^4)^, **Fresh** ^4)^, Other ^2)^, Not Sure ^3)^
Stale/Papery ^1)^	CATA	**Stale** ^4)^, **Woody** ^4)^, **Cardboard** ^4)^, **Papery** ^4)^, Other ^2)^, Not Sure ^3)^
Chemical ^1)^	CATA	**Bitter** ^4)^, **Medicinal** ^4)^, **Petroleum** ^4)^, **Skunky** ^4)^, **Rubber** ^4)^, **Salty** ^4)^, Other ^2)^, Not Sure ^3)^
Alcohol/Fermented ^1)^	CATA	**Fermented** ^4)^, **Overripe/Near Fermented** ^4)^, **Winey** ^4)^, **Whiskey** ^4)^, Other ^2)^, Not Sure ^3)^
Flavor Intensity	CATA	Mild, Pleasant, Prominent, Strong, Overbearing
		**B. Tactile Impact**
Body/Mouthfeel	CATA	**Thickness** ^4)^, Tea Like, Mouthwatering, Smooth, Grainy, Creamy, Soft, Round, Bold, Thin, **Metallic** ^4)^, **Mouth Drying** ^4)^, Oily
Body/Mouthfeel Intensity	Check-One	High, Medium, Low
		**C. Taste Basics**
Sweetness	Check-One	**Sweet** ^4)^, **Molasses** ^4)^, **Maple Syrup** ^4)^, **Brown Sugar** ^4)^, **Caramelized** ^4)^, **Honey** ^4)^, **Vanilla** ^4)^
Sweetness	Check-One	Pleasant, Unpleasant
Acidity	CATA	Crisp, Clean, Biting, Juicy, **Winey** ^4)^, Vibrant, Tart, **Citric Acid** ^4)^, Bright, Sparkling, Puckering
Acidity	Check-One	Pleasant, Unpleasant
Bitter Intensity	0–100 points scale	
Bitter	Check-One	Pleasant, Unpleasant
Umami	CATA	Mouthwatering, Complex, Tongue Coating, Savory, Long Lasting
		**D. Overall Impact**
Continuity of Taste/Flavor	Yes/No	(If evaluators choose ‘Yes’, it will move on to the below menu.)
Continuity of Taste/Flavor	CATA	Initial, Middle, Late, Finish
Aftertaste	CATA	Juicy, Clean, Persistent, Simple, Complex, Astringent, Flat, Elegant
Aftertaste	Check-One	Short, Medium, Long
Holistic/Hedonic	CATA	Aromatic, Pleasant, Balanced/**Blended** ^4)^, Harmonious, **Full/Bold** ^4)^, Lively, Stable, Irregular/Disjointed, Bland/Flat
Key Memorable Experience	Expository Text	
Unique Characteristics	Expository Text	
Balance/Amplitude	CATA	Complex, Elegant, Fullness, Satisfying, Blended, Harmonious, Balanced, Stable Through Cooling, Bland/Flat, Falls Apart Through Cooling, Disjointed, Unbalanced
Defects	CATA	**Stale** ^4)^, **Papery/Cardboard** ^4)^, **Fermented** ^4)^, **Metallic** ^4)^, **Burnt** ^4)^, **Rubber** ^4)^, **Medicinal** ^4)^, Potato, **Musty** ^4)^, Underdeveloped, **Moldy** ^4)^, **Petroleum** ^4)^, **Woody** ^4)^, Rancid, **Over Fermented** ^4)^, **Smoky** ^4)^, **Salty** ^4)^, Other ^2)^, Not Sure ^3)^
Stability (through cooling)	Yes/No	
Additional Comments	Open-ended comments	
		**E. Final Questions**
Overall Quality	0–100 points scale	
Willing to use for espresso extraction	Yes/No	
Final Comments	Open-ended comments	

^1)^ The ‘Aroma’ and ‘Flavor’ evaluations contained the same set of characteristic terms. ^2)^ The ‘Other’ selection is optional, allowing participants to provide additional characteristics if those they perceive are not on the list during the evaluation. ^3)^ The ‘Not Sure’ selection is also available to indicate that there were no defining characteristics; however, when ‘Not Sure’ was chosen, other characteristics could not be selected. ^4)^ Terms in bold are taken from the World Coffee Research Sensory Lexicon [28].

**Table 5:** Demographic information.

	Total (n = 46)	France (n = 7)	India (n = 12)	Italy (n = 7)	Republic of Korea (n = 10)	USA (n = 10)
	**Frequency (%)**	**Frequency (%)**	**Frequency (%)**	**Frequency (%)**	**Frequency (%)**	**Frequency (%)**
**Gender**						
Man	33 (71.7)	5 (71.4)	8 (66.7)	6 (85.7)	8 (80.0)	6 (60.0)
Woman	12 (26.1)	1 (14.3)	4 (33.3)	1 (14.3)	2 (20.0)	4 (40.0)
Transgender	0 (0.0)	0 (0.0)	0 (0.0)	0 (0.0)	0 (0.0)	0 (0.0)
Non-binary/non-conforming	1 (2.2)	1 (14.3)	0 (0.0)	0 (0.0)	0 (0.0)	0 (0.0)
Prefer not to respond	0 (0.0)	0 (0.0)	0 (0.0)	0 (0.0)	0 (0.0)	0 (0.0)
**Age**						
19–25	2 (4.3)	0 (0.0)	0 (0.0)	0 (0.0)	0 (0.0)	2 (20.0)
26–35	17 (37.0)	4 (57.1)	5 (41.7)	0 (0.0)	8 (80.0)	0 (0.0)
36–45	18 (39.1)	3 (42.9)	7 (58.3)	4 (57.1)	2 (20.0)	2 (20.0)
46–55	5 (10.9)	0 (0.0)	0 (0.0)	3 (42.9)	0 (0.0)	2 (20.0)
56–65	2 (4.3)	0 (0.0)	0 (0.0)	0 (0.0)	0 (0.0)	2 (20.0)
66–75	2 (4.3)	0 (0.0)	0 (0.0)	0 (0.0)	0 (0.0)	2 (20.0)
76 and above	0 (0.0)	0 (0.0)	0 (0.0)	0 (0.0)	0 (0.0)	0 (0.0)
Prefer not to respond	0 (0.0)	0 (0.0)	0 (0.0)	0 (0.0)	0 (0.0)	0 (0.0)
**Role in Coffee Industry (Check-all-that-apply)**						
Barista	20 (43.5)	6 (85.7)	2 (16.7)	0 (0.0)	9 (90.0)	3 (30.0)
Roaster	23 (50.0)	6 (85.7)	5 (41.7)	4 (57.1)	5 (50.0)	3 (30.0)
Grower	4 (8.7)	0 (0.0)	4 (33.3)	0 (0.0)	0 (0.0)	0 (0.0)
Buyer	13 (28.3)	4 (57.1)	4 (33.3)	0 (0.0)	4 (40.0)	1 (10.0)
Other	16 (34.8)	0 (0.0)	8 (66.7)	3 (42.9)	1 (10.0)	4 (40.0)

**Table 6:** Cluster composition of CLUSCATA results ^1^.

Cluster A (n = 21)		Cluster B (n = 9)	Cluster C (n = 44)				Cluster D (n = 42)				Cluster E (n = 22)		
1302 ^2^	2308	1301	1409	2402	3407	3401	1108	1204	3208	2805	1408	3102	
1303	2309	1307	1404	2403	3406	1104	1105	1205	3203	2802	2408	3109	
1305	2305	1310	1402	2409	3405	1103	1107	1208	3207	2804	1110	3106	
1306	3305	2301	1412	2412	3409	2103	1101	1203	1803	3803	1106	3110	
1304	3306	2307	1406	2411	3411	2104	2108	2204	1807	3805	1109	1201	
1309	3308	2310	1405	2407	3408	3103	2107	2205	1805	3807	1102	1206	
1308	3309	3307	1401	2406	3410	3104	2101	2203	1804	3806	2102	1207	
2303	3303	3310	1407	2401	3412	1801	2105	2208	1806	3804	2110	2201	
2304	3302	3301	1410	2410	3402	2801	3107	2207	1802	3802	2106	2206	
2302	3304		1411	2405	3404	2806	3101	3204	2807		2109	3201	
2306			1403	2404	3403	3801	3105	3205	2803		3108	3206	

^1^ CLUSCATA was conducted for all five countries, including each expert’s data from three replications. ^2^ Numbers are comprised of replication (1st digit = 1–3), country (2nd digit: 1 = USA, 2 = Italy, 3 = Republic of Korea, 4 = India, and 8 = France) and the expert within a country (3rd and 4th digit, e.g., the 1st expert in a country is coded 01).

**Table 7:** The homogeneity index by country ^1^.

	Total (n = 138)	France (n = 21)	India (n = 36)	Italy (n = 21)	Republic of Korea (n = 30)	USA (n = 30)
Homogeneity Index	0.364	0.606	0.572	0.393	0.652	0.562
Percent of experts being grouped with themselves (%)	91.3	85.7	75.0	71.4	100.0	90.0

^1^ CLUSCATA was conducted in each country for the three-replication data.

**Table 8:** The RV coefficient on sample configurations from correspondence analysis ^1^.

	France	India	Italy	Republic of Korea	USA
France	1	-	-	-	-
India	**0.828 ^2^**	1	-	-	
Italy	**0.893**	**0.820**	1	-	-
Republic of Korea	0.483	0.473	**0.701**	1	-
USA	0.338	0.388	0.570	**0.745**	1

^1^ Correspondence analysis was performed for sample configurations. ^2^ Bold indices indicate that the RV coefficient was significant (*p* < 0.05).

**Table 9:** The average overall quality scores by sample ^1^.

	Colombia	Ethiopia	Brazil	Honduras	Costa Rica	Guatemala	Brazil Cerrado	Pre- Packaged	MSD	*p*-Value
France	77.1 ^a 2^	83.2 ^a^	79.7 ^a^	76.2 ^a^	80.5 ^a^	79.8 ^a^	81.5 ^a^	61.3 ^b^	9.3752	<0.0001
India	78.1 ^a^	78.3 ^a^	73.6 ^a^	74.8 ^a^	72.7 ^a^	74.7 ^a^	73.4 ^a^	66.1 ^b^	6.5176	<0.0001
Italy	55.5 ^a^	67.0 ^a^	57.7 ^a^	54.2 ^a^	51.6 ^a^	55.8 ^a^	61.0 ^a^	27.0 ^b^	17.6620	<0.0001
Republic of Korea	57.6 ^bc^	81.0 ^a^	59.6 ^bc^	65.1 ^b^	63.1 ^b^	67.0 ^ab^	57.9 ^bc^	44.8 ^c^	15.7660	<0.0001
USA	70.1	75.4	75.2	72.5	72.9	74.5	73.9	71.7	11.6150	0.8634
Total	68.3 ^a^	77.3 ^a^	69.4 ^b^	69.3 ^b^	68.6 ^b^	70.9 ^b^	69.5 ^b^	56.0 ^c^	6.3571	<0.0001

^1^ One-way ANOVA was performed for overall quality scores within each country. ^2^ Different letters within a row indicate significant differences for the Tukey post-hoc test with minimum significant difference (MSD) (*p* < 0.05).

## Data Availability

The datasets presented in this article are not readily available because of ethical restrictions to protect participants’ privacy.

## Supplementary Materials

The following supporting information can be downloaded at: https://www.mdpi.com/article/10.3390/foods14040593/s1, Table S1: The Roasting Information.
