# Supplementary material for: Cross-Cultural Comparison of the Espresso Protocol Repeatability
Source: Foods. 2025 Feb 11;14(4):593. doi: 10.3390/foods14040593 (PMC11854300; doi:10.3390/foods14040593)
Supplement: Supplementary file 1 [file foods-14-00593-s001.zip › foods-3424895-supplementary.pdf]

**Supplementary Table S1.** The Roasting Information.

| | France | Italy | India | Korea | USA |
|---|---|---|---|---|---|
| **Roasting Equipment** | | | | | |
| Manufacturer | GIESEN | Probot | Probatino | Probat | DIEDRICH |
| Model | W6/W6A (ver.2021) | Probatino | BRZ 2 240/50 | Probatino | 1R-3N |
| Size | 6KG | 1KG | - | 1.2KG | 0.45-3.18KG (1-7lb) |
| Fuel | - | LPG | Propane | - | Propane |
| **Roasting Process** | | | | | |
| Roast Time | | 8:00±0:15 | | | |
| Roast Level[1] | #59.9±2.3 | Agtron Gourmet 76±6 (Ground coffee) | | #65 | #48.7±0.9 |
| Peak Temperature (℃) | 194.4±1.2 | 180.0±1.0 | | 192.5±1.2 | - |
| End Temperature (℃) | 212.6±1.8 | 219.0±2.0 | | - | 213.7±2.1 |
| Other | - | - | - | - | - |

[1] Color of roasted coffee bean based on SCA or Agtron number.
